# Long-term risk of heart failure in adult cancer survivors: a systematic review and meta-analysis

**DOI:** 10.1136/heartjnl-2024-324301

**Published:** 2024-08-22

**Authors:** Joshua Wong, Cheng Hwee Soh, Benjamen Wang, Thomas Marwick

**Affiliations:** 1 Baker Heart and Diabetes Institute, Melbourne, Victoria, Australia; 2 Baker Department of Cardiometabolic Health, University of Melbourne, Melbourne, Victoria, Australia; 3 Department of Cardiology, The Royal Melbourne Hospital, Melbourne, Victoria, Australia; 4 Menzies Institute for Medical Research, Hobart, Tasmania, Australia

**Keywords:** Risk Factors, Cardiomyopathy, Dilated, Cardiovascular Diseases

## Abstract

**Background:**

Cancer survivors are at increased risk of heart failure (HF). While cardiotoxicity is commonly sought at the time of cancer chemotherapy, HF develops as a result of multiple ‘hits’ over time, and there is limited evidence regarding the frequency and causes of HF during survivorship.

**Objectives:**

This systematic review sought to investigate the relationship between cardiotoxic cancer therapies and HF during survivorship.

**Methods:**

We searched the EMBASE, MEDLINE and CINAHL databases for studies reporting HF in adult survivors (≥50 years old), who were ≥5 years postpotential cardiotoxic cancer therapy. A random effects model was used to examine the associations of HF.

**Results:**

Thirteen papers were included, comprising 190 259 participants (mean age 53.5 years, 93% women). The risk of HF was increased (overall RR 1.47 (95% CI (1.17 to 1.86)). Cardiotoxic treatment, compared with cancer alone, provided a similar risk (RR of 1.46 (95% CI 0.98 to 2.16)). The overall HF incidence rate was 2.1% compared with 1.7% in the control arm—an absolute risk difference of 0.4%. In the breast cancer population ratio (11 studies), the overall HF RR was 2.57 (95% CI 1.35 to 4.90)). Although heterogeneity was significant (I^2^=77.2), this was explained by differences in patient characteristics; once multivariable analysis accounted for follow-up duration (OR 0.99, 95% CI (0.97 to 0.99), p=0.047), age (OR 1.14, 95% CI (1.04 to 1.25), p=0.003) and hypertension (OR 0.95, 95% CI (0.92 to 0.98), p<0.001), residual heterogeneity was low (I^2^=28.7).

**Conclusions:**

HF is increased in adult cancer survivors, associated with cardiotoxic cancer therapy and standard risk factors. However, the small absolute risk difference between survivors and controls suggests that universal screening of survivors is unjustifiable. A risk model based on age, cardiotoxic cancer therapy and standard risk factors may facilitate a selective screening process in this at-risk population.

**WHAT IS ALREADY KNOWN ON THIS TOPIC**

- Cardiotoxicity evaluation is often sought at time of cancer therapy.
- There is limited evidence regarding frequency and causes of heart failure during survivorship.

**WHAT THIS STUDY ADDS**

- Heart failure incidence is increased in adult cancer survivors and is associated with potentially-cardiotoxic cancer therapy as well as standard heart failure risk factors.
- The absolute risk difference between cancer survivors and controls is small.

**HOW THIS STUDY MIGHT AFFECT RESEARCH, PRACTICE OR POLICY**

- Universal screening of all cancer survivors is not warranted.
- Further work is needed to select specific populations where the pre-test risk is sufficiently high enough to justify heart failure screening.

## Background

Modern advances in cancer therapies have led to improvements in long-term survival, contributing to a rapidly growing survivorship cohort. It is estimated that there were 18.1 million cancer survivors in the USA in 2022, representing 5.4% of the population.^1^ This is expected to grow exponentially, to an estimated 22.5 million by 2032.^1^ In addition to secondary malignancies, renal impairment, endocrinopathies, mental health disorders, these survivors are susceptible to developing cardiovascular disease (CVD). A recent large prospective cohort study found that adult cancer survivors had a 42% increased risk of CVD compared with healthy controls.^2^ Heart failure (HF) accounted for the majority (52%) of CVD events.^2^ This vulnerability to HF has been described as a ‘multihit’ phenomenon, due to not only the long-term sequelae of cardiotoxicity from cancer therapies (chemotherapy, radiotherapy) but also risk factors common to cancer and CVD (ie, smoking) and premature ageing from prior cancer therapies.

Paediatric cancer survivors are 15 times more likely to develop HF compared with their healthy siblings, and this long-term research has led to the formation of HF surveillance guidelines.^3^ This contrasts with the paucity of evidence in adult cancer survivorship. In adults, most research has been focused on CV risk assessment at the time of chemotherapy, with routine surveillance echocardiograms now endorsed by recent guidelines.^4^ Observational data and population studies have suggested increased long-term CV risk in adult cancer survivors,^5 6^ but prospective studies are still lacking. Paterson *et al* demonstrated in a retrospective population-based cohort study of 2 24 016 patients that a new cancer diagnosis was independently associated with an increased risk for HF and cardiovascular death.^7^ Despite the increased risk, this has not translated to HF surveillance programmes being incorporated into cancer survivorship care. This is in part due to the ambiguity around the magnitude of risk and appropriate screening selection. Is the risk of HF significant enough to warrant universal screening or is a tailored strategy more appropriate for adult cancer survivors? Accordingly, we sought to identify and critically evaluate the prevalence of HF in adult cancer survivors who had undergone potentially cardiotoxic cancer therapy >5 years previously.

## Methods

### Search strategy

The search strategy was conducted in line with the Preferred Reporting Items for Systematic Reviews and Meta-Analyses (PRISMA) guidelines and archived at Open Science Framework (https://osf.io/beq7s/). The electronic databases EMBASE, MEDLINE and CINAHL were searched systematically by an information specialist to include published articles from date of inception to 22 January 2024. Key search terms included ‘cancer survivors’, ‘cancer therapy’, ‘heart failure’ and their variations. The search strategy is listed in detail in online supplemental table 1. The term ‘survivors’ was used as the MeSh (medical subject headings) for ‘cancer survivors’ was only available from 2018 onwards.

10.1136/heartjnl-2024-324301.supp1 Supplementary data

### Study selection

**Figure 1:**
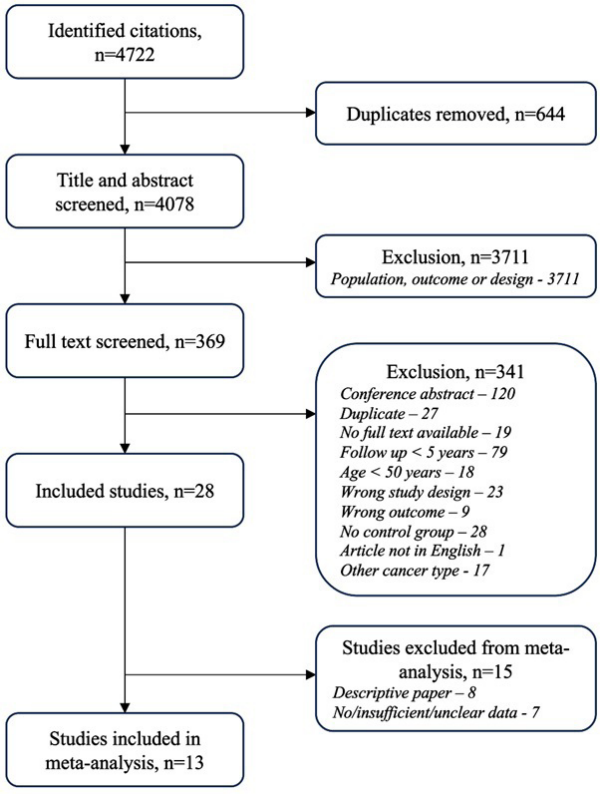
Study selection process.

The selection process is summarised in figure 1. Studies which involved long-term follow-up (>5 years) of cancer patients, and which reported HF as an outcome were selected for review. The age of adult survivors was confined to ≥50 years to avoid including survivors of childhood cancers. Cancer therapy included chemotherapy, radiotherapy or immunotherapy. Cardiotoxic therapies were defined as anthracyclines, human epidermal receptor-2 antagonists, vascular endothelial growth factor inhibitors, tyrosine kinase inhibitors, chest radiotherapy (RT) and immune checkpoint inhibitors. Studies had to provide a control group (either cancer survivors without potentially cardiotoxic therapy or healthy controls). Eligible study designs included randomised control trials, cohort studies and case–control studies. All cancer types were included. Unpublished manuscripts or conference abstracts were deemed ineligible for inclusion. Studies were not restricted by language. Title and abstract screening were performed by one reviewer (JW) and confirmed by a second (BW). Conflicts were reviewed and resolved via consensus or review with a third investigator (T.M.)

### Data extraction

The following parameters were extracted: study type, year of publication, patient demographics, sample size, known risk factors for HF (diabetes, obesity, chronic renal disease, smoking history, hypertension and hypercholesterolemia), comorbidities, medications, cancer type and therapy, follow-up duration. Of the 28 included studies, 8 were excluded from meta-analysis as they were descriptive papers and seven had insufficient data.

### Study outcomes

The study outcomes were incident HF, left ventricular ejection fraction(LVEF), diastolic dysfunction, CV events and death in cancer survivors.

### Statistical analysis

The combined weighted prevalence of HF was calculated using a random effect restricted maximum likelihood (REML) model. Between-study heterogeneity was assessed with I^2^ statistics. The effect size of the meta-analysis was reported in risk ratio (RR), along with a 95% CI. Forest plots were also generated to visualise the effect size.

The impact of other factors on the risk difference in the HF development between cancer survivors and controls was explored individually using meta-regression. The factors included in the meta-regression were age at diagnosis of cancer, follow-up duration, sex, smoking status, cancer treatment and diagnosed comorbidities, including diabetes, hypertension, hypercholesteremia and obesity. A multivariable meta-regression analysis was also performed with variables selected via a stepwise-backward elimination process. The outputs were reported in OR and the corresponding 95% CI.

The risk of bias was assessed via Trim and Fill test for publication bias and an Egger regression test for small-study effect. Statistical tests were set as twosided, and significance was defined as a p value of <0.05. All analyses were performed using the Stata V.18.0 statistical software.

## Results

### Study characteristics

The PRISMA search strategy (figure 1) rendered a total of 4722 studies. After removing duplicates, a total of 4078 articles were screened for eligibility via title and abstracts, and subsequently 369 articles were screened in full text. Thirteen papers were included in the review and meta-analysis, comprising of 190 259 participants. The study population included 11 publications related to breast cancer cohorts and two related to lymphoma, published between 2014 and 2022. Papers were noted to commonly distinguish the study design in two populations: cancer and potentially cardiotoxic therapy compared with cancer alone and cancer and potentially cardiotoxic therapy compared with healthy controls. Both study designs assess the degree to which cancer and potentially cardiotoxic therapy impact the development of HF. Incident HF was defined in the majority of studies by ICD-9 or ICD-10 diagnosis codes of ‘HF’ or ‘congestive HF’. In a minority of studies HF was defined by clinical assessment. These diagnoses were made across both inpatient and outpatient settings. Two papers were included from the same author as the study design and selection criteria for the two studies were different.^8 9^ The first included women with breast cancer who received treatment between 1970 and 2007 (n=700) and examined incidence of CVD against healthy controls.^8^ The second included women treated for stage I-III breast cancer who had been free of disease for >5 years (n=2196) and assessed LVEF against cancer controls.^9^

### Baseline characteristics

The baseline demographic data of included studies (table 1) showed a mean age of 53.5 years, and a female predominance (93%), both of these findings reflecting and over-representation of breast cancer.

**Table 1:** Baseline characteristics

Study	Banke *et al* 21	Boerman *et al* 8	Boerman *et al* 9	Chien *et al* 29	Chung *et al* 10	Franchi *et al* 17	Fumoleau *et al.* 11	Ganz *et al* 16	Kwan *et al* 19	Lee *et al* 18	Puckett *et al* 12	Ociet *et al* 20	Juul *et al* 13
Year	2019	2013	2017	2016	2020	2020	2006	2017	2022	2019	2021	2021	2022
Country	Denmark	Netherlands	Netherlands	Taiwan	South Korea	Italy	France	USA	USA	China	USA	USA	Denmark
Total (n)	8812	2196	700	4845	11 863	18 624	3577	407	88 838	3489	200	11 098	11 099
Follow-up (years)	5.4	9	10	5.29	5.6	5.88	7	8.8	7	5.2	11.5	5	9.5
Incident heart failure (n)	111	79	4	151	112	308	5	15	1262	206	2	89	365
Age (years)	52.35	53.66	51.25	50.99	53.82	55	52.58	58.3	61.1	49.37	50.14		81
Male (%)	0	0	0	0	0	0	0	0	0	0	0	54.78	48.86
Hypertension (%)	7.62	12	10.9	17.7	26	29.97		20.9	42	18.43	26.5		
Diabetes mellitus (%)	2.17	3.3	2.1	8.97	7.5	6.93		3.4	13	6.22	15		11.3
Dyslipidaemia (%)		4.83	4.4	2.8	31.3			11.05	41.33		36		
Chronic kidney disease (%)						0.13			15.75		0.5		
Arrhythmia (%)				1.44		0.57			0.95				
Atrial fibrillation (%)	0.67		0.43								2.5		12.39
Ischaemic heart disease (%)	2.66		0.86	3.05					0.67				16.64
Acute myocardial infarction (%)	0.5												
Heart failure (%)		3.6	0						0				7.43
Smoking (%)									32.18		30.5	8.82	
Chronic obstructive pulmonary disease (%)	1.42												9.31
Cerebrovascular disease (%)		4.64	2.14										
Angiotensin-converting enzyme (ACE) inhibitors/angiotensin receptor blocker (ARB)			2.86	13.73									
Beta-blocker (%)			2.14	21.32									
Anti-platelet agent (%)			0.07	9.39									
Statin (%)			1.71	8.57									
Diuretics (%)				19.11									
Post menopausal (%)	50.4						57.25		75.53				
Surgery (%)	62.40			76.47		100	98.83						
Radiotherapy (%)	80.91		0.42	27.8	71.61		96.14						
Left breast radiation (%)			24.43				49.76		5.42				
Chemotherapy (%)	5.27		20.28		47.57				7.01				
Anthracycline (%)			20.28	70.98	47.57		71.37		4.63				
Hormone therapy				0.38	71.9		44.03		8.86		0.29		
HER2-receptor antagonist (%)			1.86		14.48				1.71				
Left breast (%)			24.43				51.41				54.5		
Right breast (%)							47.30						

Comorbidities and cancer therapies were selected if they were reported in more than three studies, 15 conditions met these requirements—diabetes, hypertension, dyslipidaemia, ischaemic heart disease (IHD), chronic kidney disease (CKD), arrhythmia, atrial fibrillation, smoking and various cancer treatments (table 1). The prevalence of diabetes ranged from 2% to 13% (weighted mean 10%), hypertension ranged from 6.8% to 42% (weighted mean 34%) and dyslipidaemia ranged from 2.8% to 46% (weighted mean 39%).

Therapies with cardiotoxic potential were identified in all papers—anthracycline,^10–13^ trastuzumab,^14–17^ anthracycline+RT,^18^ anthracycline+HER2 antagonist+RT^19^ and chemotherapy (unspecified)+RT.^8 9 20^ Information regarding dosage of chemo/radiotherapy was highly variable and absent in the majority of studies, therefore, was not extracted.

### Effect on LVEF

**Figure 2:**
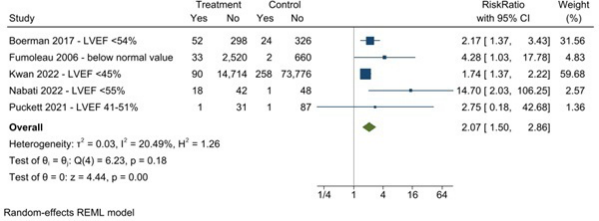
Association of cancer survivorship with reduction of left ventricular ejection fraction (LVEF).

Five breast cancer studies were included in the analysis of reduced LVEF as an outcome. There was a significant association between potential cardiotoxicity therapies and reduced LVEF, compared with the control group (RR 2.07 (CI 95% 1.50 to 2.86)) (figure 2), with a low heterogeneity (I²=20.49%). In a meta-regression, there was no significant association of reduced LVEF in follow-up (table 2).

**Table 2:** Associations of reduced LVEF

Univariable analysis					
	Studies reported	B	Overall effect size	P value	Residual heterogeneity
Follow-up	5	−0.0148822	0.985, 95%CI (0.964 to 1.007)	0.1745	58.1
Age	5	−0.0589756	0.943, 95%CI (0.799 to 1.112)	0.4837	40.28
Hypertension	4	0.0228663	1.023, 95%CI (0.94 to 1.113)	0.5953	62.41
Diabetes	4	0.0691632	1.072, 95%CI (0.853 to 1.346)	0.5527	63.22
Dyslipidaemia	3	−0.0080549	0.992, 95%CI (0.977 to 1.007)	0.2991	0
Left breast radiotherapy	3	0.0170695	1.017, 95%CI (0.996 to 1.039)	0.1188	0
Anthracycline	3	0.0136185	1.014, 95%CI (0.996 to 1.032)	0.1283	0
Hormone therapy	3	0.02054	1.021, 95%CI (0.988 to 1.055)	0.2161	0

LVEF, Left Ventricular Ejection Fraction.

### Effect on diastolic dysfunction, CV events and death

Three breast cancer studies reported diastolic dysfunction as an outcome.^9 12 19^ Three studies reported cardiac events^9 12 18^ and two described CV mortality.^11 21^ These outcomes were not included in the meta-analysis due to the limited data available.

### Overall HF incidence

**Figure 3:**
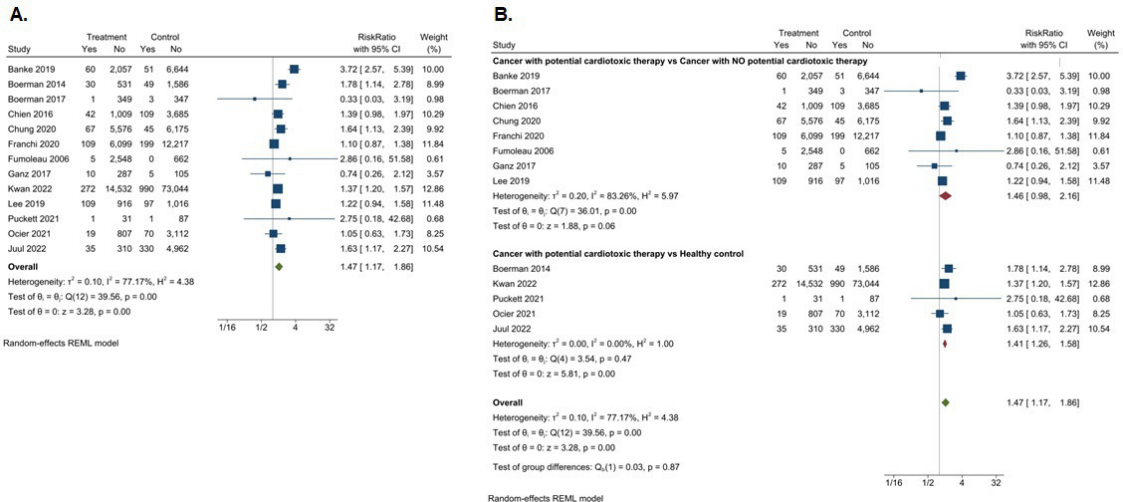
Association of cancer survivorship with incident HF; (A) incident HF in all cancer survivors, (B) incident HF in subgroups of cancer survivors. HF, heart failure; REML, random effect restricted maximum likelihood.

The cumulative incident HF rate was 2.1% in patients with previous cancer, compared with the 1.7% in the control group, with the average follow-up time ranging from 5 to 11.5 years. There was a modest positive association between cancer with potentially cardiotoxic therapy and HF in adult cancer survivors, evidenced by an overall RR of 1.47 (95% CI 1.17 to 1.86) (figure 3A). There was significant heterogeneity between the studies (I²=77.2%).

There was also a modest positive association between cancer and potentially cardiotoxic treatment and HF, compared with cancer alone (RR of 1.46 (95% CI 0.98 to 2.16)) (figure 3B). The association of cancer with potentially cardiotoxic treatment was stronger when compared with healthy controls (RR of 1.41 (95% CI 1.26 to 1.58)). In the breast cancer population (n=11 studies), the overall HF RR was 2.57 (95% CI 1.35 to 4.90). Wide CIs for the associations are likely secondary to the small sample size and significant heterogeneity between studies.

Determinants of heterogeneity were assessed via an exploratory meta-regression (table 3). Seventeen variables were investigated in the meta-regression: follow-up, age, hypertension, diabetes, dyslipidaemia, IHD, CKD, arrhythmia, atrial fibrillation, smoking and cancer treatment. Multivariable analysis demonstrated significant effect sizes for follow-up duration (OR 0.99 (95% CI 0.97 to 0.99), p=0.047), age (OR 1.14 (95% CI 1.04 to 1.25), p=0.003) and hypertension (OR 0.95 (95% CI 0.92 to 0.98), p<0.001). Once these variables were considered, residual heterogeneity was low (I²=28.7%), confirming that initial heterogeneity is explained by patient characteristics.

**Table 3:** Associations of incident HF

Univariable analysis					
	Studies reported	B	Overall effect size	P value	Residual heterogeneity
Follow-up	13	−0.0029544	0.997, 95%CI (0.986 to 1.008)	0.6022	77.23
Age	10	−0.0307534	0.97, 95%CI (0.88 to 1.068)	0.5325	79.38
Hypertension	10	−0.0180449	0.982, 95%CI (0.96 to 1.005)	0.1273	71.9
Diabetes	11	−0.0331442	0.967, 95%CI (0.903 to 1.036)	0.3419	72.92
Dyslipidaemia	6	0.0029563	1.003, 95%CI (0.997 to 1.009)	0.2981	0
Ischaemic heart disease	5	0.0011343	1.001, 95%CI (0.958 to 1.046)	0.9597	91.05
Chronic kidney disease	3	0.014343	1.014, 95%CI (0.997 to 1.032)	0.1054	0
Atrial fibrillation	4	−0.0040304	0.996, 95%CI (0.894 to 1.11)	0.9419	54.05
Arrhythmia	3	0.3414124	1.407, 95%CI (0.872 to 2.27)	0.162	0
Smoking (past or current)	3	0.0361338	1.037, 95%CI (0.974 to 1.103)	0.2531	0
Surgery	4	−0.0267093	0.974, 95%CI (0.947 to 1.002)	0.0635	68.4
Radiotherapy	5	0.0154967	1.016, 95%CI (0.994 to 1.038)	0.1607	65.22
Left breast radiotherapy	3	0.007131	1.007, 95%CI (0.934 to 1.086)	0.8527	42.94
Chemotherapy	3	−0.0083271	0.992, 95%CI (0.952 to 1.034)	0.693	95.95
Anthracycline	5	0.0013522	1.001, 95%CI (0.996 to 1.007)	0.6081	0
Endocrine therapy	5	0.0026365	1.003, 95%CI (0.997 to 1.009)	0.3874	0
HER2 receptor antagonists	3	0.0330386	1.034, 95%CI (0.91 to 1.174)	0.6113	34.21

Multivariable Analysis					
	Studies reported	B	Overall effect size	P value	Residual heterogeneity
Follow up	9	−0.0141762	0.986, 95%CI (0.972 to 0.999)	0.047	28.73
Age	9	0.1311866	1.14, 95%CI (1.044 to 1.245)	0.003	
Hypertension	9	−0.052045	0.949, 95%CI (0.924 to 0.975)	<0.001	

HF, heart failure.

**Figure 4:**
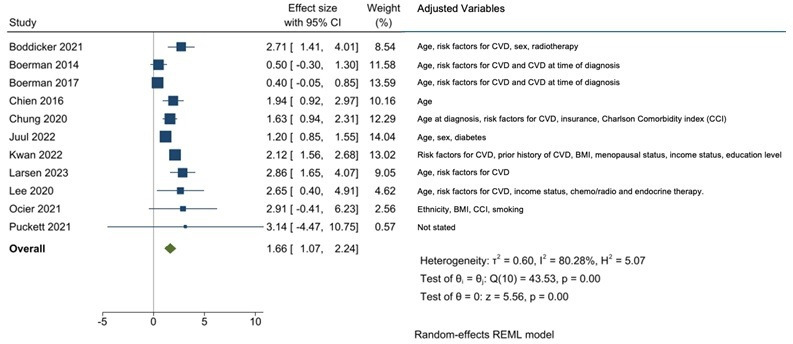
Association of exposure to potentially cardiotoxic therapy with incident HF, independent of other risk factors. BMI, body mass index; CVD, cardiovascular disease; HF, heart failure; REML, random effect restricted maximum likelihood.

The significant association between potential cardiotoxic therapy and HF persisted after adjustment for age, sex, risk factors for CVD and/or pre-existing CVD (figure 4).

### Time course

**Figure 5:**
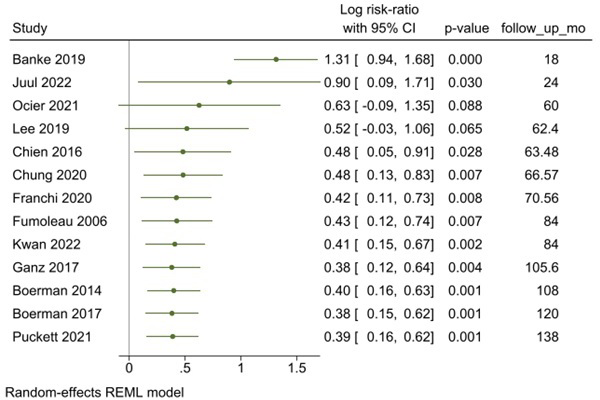
Annualised HF risk in cancer survivors. HF, heart failure; REML, random effect restricted maximum likelihood.

On univariable and multivariable analyses, follow-up duration demonstrated a diminishing risk of HF as time increased. Annualising HF risk accounts for follow-up duration and further establishes this finding (figure 5). These findings also demonstrate that studies with shorter follow-up have the widest CIs.^13 21^

### Publication bias

The funnel plot (online supplemental figure 1B) demonstrated no publication bias for assessment of HF. This was further confirmed using Duval and Tweedie’s trim-and-fill analysis in which no study was removed (trim) or imputed (fill) due to publication bias (online supplemental figure 1C). Egger’s test also confirmed there were no small study effects in the assessment of HF (p value=0.6973) (online supplemental figure 1D).

## Discussion

There are multiple key findings from this systematic review and meta-analysis on late-onset HF in adult cancer survivors. Left ventricular (LV) dysfunction occurs during survivorship. HF incidence is higher among adult cancer survivors who have undergone potentially cardiotoxic therapy compared with those without potentially cardiotoxic therapy or healthy controls. Variables such as follow-up duration, age and hypertension are independently associated with incident HF, with follow-up duration demonstrating diminishing risk as duration increases.

### HF in cancer survivorship

The relationship between cardiotoxic cancer treatments and the development of HF has been well established in the early treatment phase. However, less attention has been given to quantifying long-term HF risk in adult cancer survivors. A recent case–control study by Larsen *et al* of adult cancer survivors^22^ demonstrated the cumulative incidence of HF in cancer survivors postanthracycline was 7.4% over 15 years—more than double the risk of matched controls. A 2013 meta-analysis on the incidence and predictors of anthracycline cardiotoxicity over a median follow-up of 9 years found a 6% (95% CI 3% to 9%) incidence of clinically overt cardiotoxicity and 18% (95% CI 12% to 24%) incidence of subclinical cardiotoxicity.^23^ Despite this population being at increased risk of HF, there is no targeted screening programme for adult cancer survivors. The 2022 European Society of Cardiology Guidelines on cardio-oncology provides Class IIb/Level C recommendations that 5 yearly echocardiographic screening *may* be considered in adult cancer survivors with moderate–high risk.^4^ Because of the lack of an early identification process, cancer survivors may be at risk of the significant burden and cost of symptomatic HF.

### The rationale of HF screening in cancer survivors

The development of HF in survivors satisfies a number of requirements of a screening programme. HF is an important diagnosis, with a well-understood natural history, detectable in an early stage and having accepted treatments.^24^ The selection of survivors for such a screening process would be dependent on their clinical risk, together with HF-specific markers such as echocardiography or measurement of natriuretic peptides.^25^ Echocardiographic abnormalities (including LV remodelling, diastolic dysfunction and reduced systolic function) are the cornerstones for recognition of ‘stage B’ (SBHF), and these patients are five times more likely to develop clinical HF compared with controls having normal LV function.^26^

SBHF is treatable with cardioprotective strategies based on neurohormonal blockade. This has been shown to be effective in preventing the progression of asymptomatic LV dysfunction to symptomatic HF, although in trials mainly involving ischaemic HF.^27^ However, the 2022 ESC Cardio-Oncology Guidelines judged that the usefulness of treatment of asymptomatic mild CTRCD with ACE-inhibitors/ARB and/or beta-blockers was not well supported by evidence or opinion (class IIb).^4^ In reference to the population of interest in this systematic review—adult cancer survivors at risk of late-onset HF—there are no current data on management of SBHF in this specific group.

### Who to screen?

The selection of patients is a critical step in screening—such a programme would require the disease to have at least a moderate prevalence in the study population to be effective. Indiscriminate screening of low-risk patients carries the risk of a high number of false-positive results, which is not cost-effective. The strongest association of HF is age; the prevalence of echocardiographically-defined SBHF is 13% in asymptomatic communities >65 years old.^28^ In our study population of adult cancer survivors, the overall HF incidence rate was 2.1% compared with 1.7% in the control arm—an absolute risk difference of 0.4%. There were also no cancer-related/treatment-related predictors that were associated with the outcome. These findings suggest that cancer alone is not a significant enough risk factor to warrant universal HF screening in survivors and a tailored strategy is required. As age, hypertension and follow-up were independently associated with HF in this study, combining these features may aid in forming a subpopulation in which screening is valuable, for example, cancer survivors ≥65 years with HF risk factors. No such screening recommendation has been made in the general population.

### Limitations

There are several limitations of this work. First, the results of a systematic review are inevitably constrained by the material available in the individual papers. There was significant variability in the reporting of CV risk factors, CVD and CV medications among the various studies. Granular data such as dosage of chemo/radiotherapy were also unavailable in the majority of papers. Natriuretic peptides may also be increased in SBHF, but natriuretic peptide levels were not recorded in the studies included in this analysis. Second, there was significant heterogeneity in the studies, primarily stemming from different populations of interest. Breast cancer and haematological malignancies have significant variation in their predominant gender, age and associated comorbidities. This, combined with a relatively small sample size, contributed to wide CIs for the associations detected in this study. Third, studies had a variable duration, and this makes it difficult to compare the incidence of HF. We tried to overcome this by comparing annualised HF risk (figure 5), but the underlying assumption of linearity of risk may be unjustified.

## Conclusion

As the cancer survivorship population continues to grow, the impact of late-onset HF can be expected to increase. This systematic review and meta-analysis demonstrates that incident HF is increased in adult cancer survivors. Variables such as age, hypertension and follow-up duration are independently associated with incident HF. However, the risk difference between survivors and controls is small and not sufficient to warrant universal screening. Further work is needed to select specific target populations among whom pretest risk is sufficiently high to justify a screening strategy.

## Data Availability

Data are available upon reasonable request. Proposals for collaboration from appropriate parties are welcomed. Interested scientists should contact the senior author (tom.marwick@baker.edu.au)
