# Supplementary material for: Long-term risk of heart failure in adult cancer survivors: a systematic review and meta-analysis
Source: Heart. 2024 Aug 22;110(19):1188–95. doi: 10.1136/heartjnl-2024-324301 (PMC11420760; doi:10.1136/heartjnl-2024-324301)
Supplement: Supplementary data [file heartjnl-2024-324301supp001.pdf]

Supplementary Table 1: Search Strategy

**Ovid MEDLINE**		
#	Searches	Results
1	heart failure/ or heart failure, diastolic/ or heart failure, systolic/	151692
2	heart failure.ti,ab.	216614
3	cardiac failure.ti,ab.	12949
4	cardiogenic shock.ti,ab.	15576
5	cardiomyopathy.ti,ab.	84501
6	left ventricular failure.ti,ab.	2309
7	systolic dysfunction.ti,ab.	9990
8	diastolic dysfunction.ti,ab.	11810
9	1 or 2 or 3 or 4 or 5 or 6 or 7 or 8	348376
10	exp Antineoplastic Agents/	1270673
11	exp Radiotherapy/	210814
12	chemotherapy*.ti,ab.	453375
13	radiotherapy*ti,ab.	210985
14	(immunotherapy*adj3 cancer).ti,ab.	26774
15	antineoplastic agent*ti,ab.	5972
16	anticancer agent*.ti,ab.	24042
17	10 or 12 or 13 or 14 or 15 or 16	1805688
18	Cancer Survivors/	9722
19	Survivors/	31453
20	(Survivor*adj3 cancer).ti,ab.	28781
21	cancer patient*.ti,ab.	248011
22	18 or 19 or 20 or 21	294053
23	9 and 17 and 22	1230
**Ovid EMBASE**		
#	Searches	Results
1	exp heart failure/	702023
2	exp systolic heart failure/	20957
3	exp diastolic heart failure/	17087
4	heart failure.ti,ab.	373786
5	cardiac failure.ti,ab.	22014
6	cardiogenic shock.ti,ab.	22972
7	cardiomyopathy.ti,ab.	139478
8	left ventricular failure.ti,ab.	3677
9	systolic dysfunction.ti,ab.	19952
10	diastolic dysfunction.ti,ab.	24418
11	1 or 2 or 3 or 4 or 5 or 6 or 7 or 8 or 9 or 10	847967
12	antineoplastic agent/	379735
13	Radiotherapy/	211598
14	chemotherap*ti,ab.	749073
15	radiotherapy*ti,ab.	341212
16	(immunotherapy*adj3 cancer).ti,ab.	37794
17	antineoplastic agent*ti,ab.	7762
18	anticancer agent*ti,ab.	30324
19	12 or 13 or 14 or 15 or 16 or 17 or 18	1375109
20	exp cancer survivor/	35703
21	Survivor/	72281
22	(Survivor*adj3 cancer).ti,ab.	44024
23	cancer patient*.ti,ab.	408682
24	20 or 21 or 22 or 23	515129

25	11 and 19 and 24	3016
**CINAHL Complete (EBSCO)**		
1	(MH "Heart Failure+")	49953
2	heart failure	80578
3	heart failure, diastolic	833
4	heart failure, systolic	1967
5	cardiac failure	6338
6	cardiogenic shock	5420
7	cardiomyopathy	22775
8	left ventricular failure	2783
9	systolic dysfunction	3466
10	diastolic dysfunction	3348
11	1 or 2 or 3 or 4 or 5 or 6 or 7 or 8 or 9 or 10	107678
12	(MH "Antineoplastic Agents+")	142111
13	(MH "Radiotherapy+")	42857
14	(MH "Chemotherapy, Cancer+")	40629
15	(MH "Immunotherapy+")	58886
16	chemotherapy	102355
17	radiotherapy	78038
18	immunotherapy	25681
19	antineoplastic agent	84629
20	anticancer agent	1884
21	12 or 13 or 14 or 15 or 16 or 17 or 18 or 19 or 20	315387
22	(MH "Cancer Survivors")	13796
23	cancer survivors	24795
24	survivors	63256
25	(MH "Cancer Patients")	53382
26	cancer patient	181872
27	22 or 23 or 24 or 25 or 26	181872
28	11 and 21 and 27	476
**Total Search (Ovid MEDLINE + Ovid EMBASE + CINAHL)**		**4722**

Supplementary Figure 1. Evaluation of publication bias –

A. **In assessment of LVEF,**

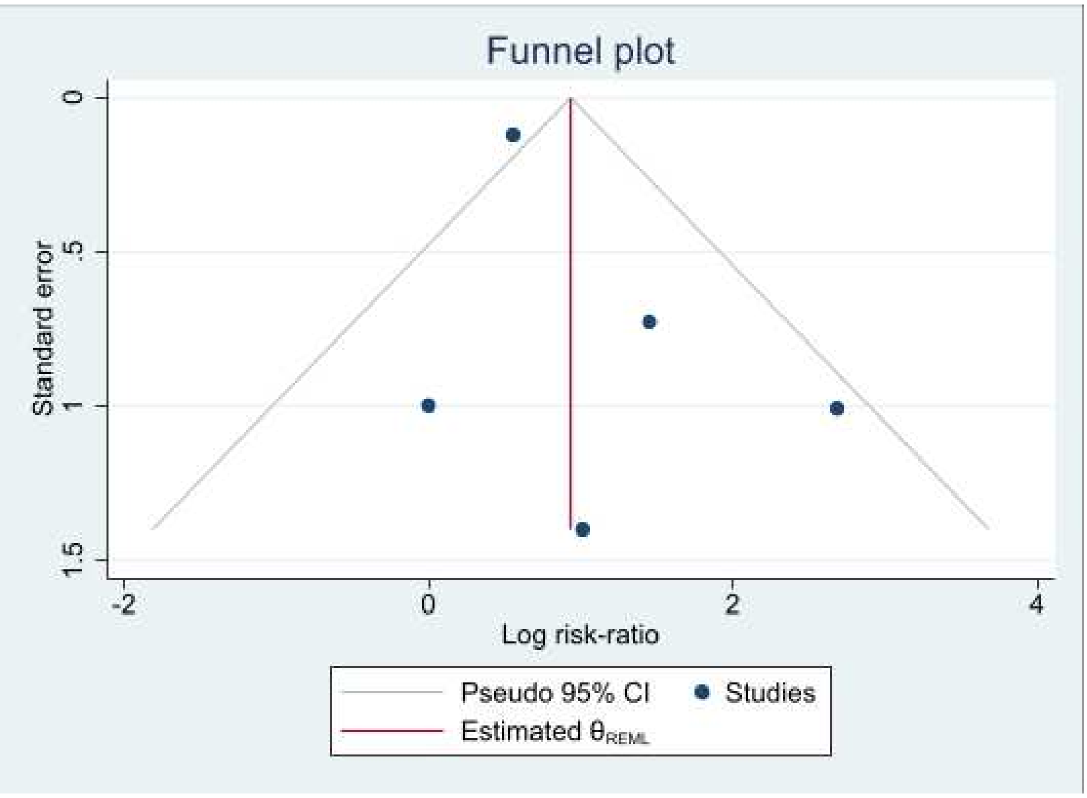

B. In assessment of HF.

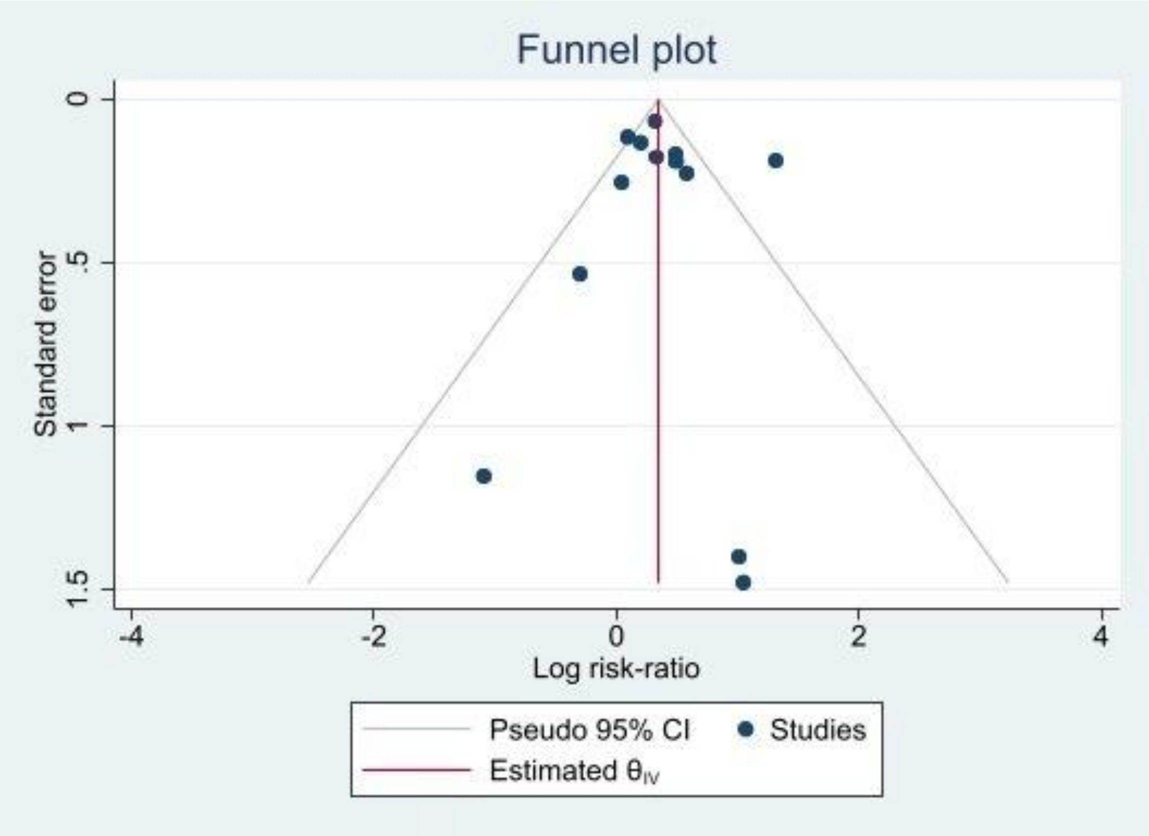

C. Evaluation of small study effect –

```
  Effect-size label: Log risk-ratio
        Effect size: _meta_es
          Std. err.: _meta_se

Regression-based Egger test for small-study effects
Random-effects model
Method: REML

H0: beta1 = 0; no small-study effects
            beta1 =     -0.24
      SE of beta1 =     0.620
                z =     -0.39
       Prob > |z| =    0.6973
```

D. Duval and Tweedie's trim and fill analysis for publication bias –

```
. meta trimfill

  Effect-size label: Log risk-ratio
        Effect size: _meta_es
          Std. err.: _meta_se

Nonparametric trim-and-fill analysis of publication bias
Linear estimator, imputing on the left

Iteration                              Number of studies =     13
  Model: Random-effects                         observed =     13
 Method: REML                                   imputed =      0

Pooling
  Model: Random-effects
 Method: REML
```

Studies	Log risk-ratio	[95% conf.	interval]
Observed	0.388	0.156	0.620
Observed + Imputed	0.388	0.156	0.620
